# Modulation of brain oscillations by continuous theta burst stimulation in patients with insomnia

**DOI:** 10.1038/s41398-025-03605-y

**Published:** 2025-10-17

**Authors:** Ximei Zhu, Lin Jiang, Le Shi, Fali Li, Qingqing Yang, Mingyue Zhang, Yinjiao Li, Qiuxuan Yu, Jie Chen, Xuejiao Gao, Zhong Wang, Yong Wang, Peng Xu, Lin Lu, Jiahui Deng

**Affiliations:** 1 Peking University Sixth Hospital, Peking University Institute of Mental Health, NHC Key Laboratory of Mental Health (Peking University), National Clinical Research Center for Mental Disorders (Peking University Sixth Hospital), Beijing, China; 2 The Clinical Hospital of Chengdu Brain Science Institute, MOE Key Lab for Neuroinformation, School of Life Science and Technology, University of Electronic Science and Technology of China, Chengdu, China; 3 Brain-Computer Interface & Brain-Inspired Intelligence Key Laboratory of Sichuan Province, University of Electronic Science and Technology of China, Chengdu, China; 4 Institute of Basic Medical Sciences, Chinese Academy of Medical Sciences and Peking Union Medical College, Beijing, China; 5 Department of Rehabilitation Medicine, Zhujiang Hospital, Southern Medical University, Guangzhou, China; 6 Peking-Tsinghua Center for Life Sciences and PKU-IDG/McGovern Institute for Brain Research, Peking University, Beijing, China; 7 National Institute on Drug Dependence and Beijing Key Laboratory of Drug Dependence, Peking University, Beijing, China

**Keywords:** Psychiatric disorders, Physiology

## Abstract

Continuous theta burst stimulation (cTBS) induces long-lasting depression of cortical excitability in motor cortex. In the present study, we explored the modulation of cTBS on resting state electroencephalogram (rsEEG) during wakefulness and subsequent sleep in patients with insomnia disorder. Forty-one patients with insomnia received three sessions active and sham cTBS in a counterbalanced crossover design. Each session comprised 600 pulses over right dorsolateral prefrontal cortex. Closed-eyes rsEEG were recorded at before and after each session. Effects of cTBS in subsequent sleep were measured by overnight polysomnography screening. Power spectral density (PSD) and phase locking value (PLV) were used to calculate changes in spectral power and phase synchronization after cTBS during wakefulness and subsequent sleep. Compared with sham cTBS intervention, PSD of delta and theta bands were increased across global brain regions with a cumulative effect after three active cTBS sessions. PLV of delta and theta bands were enhanced between stimulated frontal area and occipital areas. Efficiency of information communication within frontal-occipital networks was consistently improved through three active sessions. Increased theta power during wakefulness was positively related with that during the first sleep cycle. Active cTBS significantly enhanced the spectral power of delta and theta bands during wakefulness, with a cumulative effect observed over time. This modulation also extended to influence theta power during subsequent sleep onset period. Collectively, these findings provide a robust theoretical foundation for further investigating the therapeutic potential of long-term cTBS in the treatment of insomnia disorders.

## Introduction

Sleep-wake transition process is characterized by dynamic changes in both frequency and amplitude of brain oscillations [1]. The transition from wakefulness to sleep is associated with characteristic electrophysiological patterns, which included decreased of high frequency (beta and gamma) and increased of low frequency (delta and theta) electroencephalogram (EEG) power [2]. Alpha activity increases during drowsiness or relaxation with eyes closed. From drowsiness transitions into sleep, alpha power gradually gives way to slower delta and theta activity [3]. As reviewed by Colombo et al. [4], EEG studies during wakefulness consistently revealed sustained elevations of spectral power within the beta and gamma bands among patients with insomnia. During sleep onset period, several studies have suggested that spectral power of delta band in patients with insomnia is decreased globally in non-rapid eye movement (NREM) sleep than that in healthy people, while spectral power of beta band is increased [5–7]. Slow wave activity in first sleep cycle related with sleep quality [8]. In addition, cortical hyperarousal already exists during wakefulness; however, its effects are more pronounced during the early stages of sleep and during sleep maintenance. Collectively, these findings demonstrate that insomnia is characterized by cortical hyperarousal during wakefulness and sleep [9]. Chronic insomnia has been shown to induce dysregulation of the inflammatory process, which subsequently affects the spectral power of EEG signals and exacerbates cortical hyperarousal [10, 11].

Accumulating evidences has demonstrated that brain stimulation can modulate brain oscillations. Transcranial magnetic stimulation (TMS), a non-invasive technique, involves the application of sequences of magnetic pulses with varying patterns and frequencies to induce modulations in brain oscillations [12]. The primary explanation underlying the effects of TMS posits that TMS can modulate synaptic plasticity and the strength of connections between brain areas [13]. A variety of TMS protocols have been developed to reliably potentiate or suppress cortical activity in stimulated brain region [14]. For instance, a 1 Hz repetitive TMS (rTMS) applied over the right dorsolateral prefrontal cortex (dlPFC) for two weeks has been found to improve sleep architecture compared to pharmacotherapy and psychotherapy [15]. More recently, continuous theta burst stimulation (cTBS), as a pattern of TMS stimulation, was shown to be effective at inducing depression of cortical excitability in motor cortex for up to 60 min [16]. A recent study suggested that the cTBS significantly improved sleep efficiency, and reduced the NREM latency during subsequent night in healthy people [17]. Another study reported that one session of cTBS significantly altered functional connectivity within DMN, and improved sleep quality in patients with insomnia [18]. Despite its increasingly widespread experimental use, the precise mechanisms by which cTBS exerts its inhibitory effects on endogenous EEG activity remain unclear. We hypothesize that cTBS can modulate cortical activity within the targeted brain regions, and that this modulatory effect may subsequently influence sleep.

In the present study, we investigated the modulation of brain oscillations by cTBS in patients with insomnia. We designed a within-subject crossover design to evaluate the modulation of cTBS on resting state EEG (rsEEG) during wakefulness. Furthermore, we sought to investigate the sustained influence on subsequent sleep, to elucidate the mechanisms underlying the observed effects of cTBS in sleep of insomnia patients.

## Methods

### Participants

Participants were recruited from Peking University Sixth Hospital and local community clinics through advertisements between June and December 2021. The sample size was determined using G*Power software (version 3.1.9.2; Heinrich-Heine-Universität Düsseldorf, Düsseldorf, Germany) to ensure sufficient statistical power for paired two-tailed *t*-tests. The calculations were based on medium effect size (d = 0.5), significance level (α = 0.05), and a desired power (1−β = 0.9). Accordingly, the total sample size required was approximately 44 participants. Inclusion criteria are as follows: (1) aged 18–65 years; (2) primary insomnia diagnosed by DSM-IV; (3) stable medication or no medication for the past 2 weeks; (4) right-handed. Exclusion criteria are as follows: (1) another mental disorder meets the DSM-IV criteria (except for insomnia disorder); (2) polysomnographic (PSG) of first adaptation night indicated sleep apnea (Apnea-Hypopnea Index‌ > 15), periodic limb movement disorder, or other sleep disorder; (3) unstable physical diseases, or history of epilepsy; (4) pregnant or lactation; (5) history of electroconvulsive therapy, TMS, or transcranial direct current stimulation; (6) history of a cochlear implant, cardiac pacemaker, and any brain devices or/and implants; (7) hyperalgesia, skin damage or inflammation in the stimulated area; and (8) night shift workers during the last year.

Forty-five people with primary insomnia were recruited in the study. Three participants were excluded due to the apnea-hypopnea index >15 on the first adaptation night. One participant was excluded due to low EEG recording quality. Thus, the data presented here include 41 participants. Our study was approved by the Ethics Committee of the Medicine Faculty of the University of Peking Sixth Hospital. Participants gave their written informed consent after the nature and possible consequences of the studies were explained and received a financial compensation.

### Experimental protocol

**Fig. 1:**
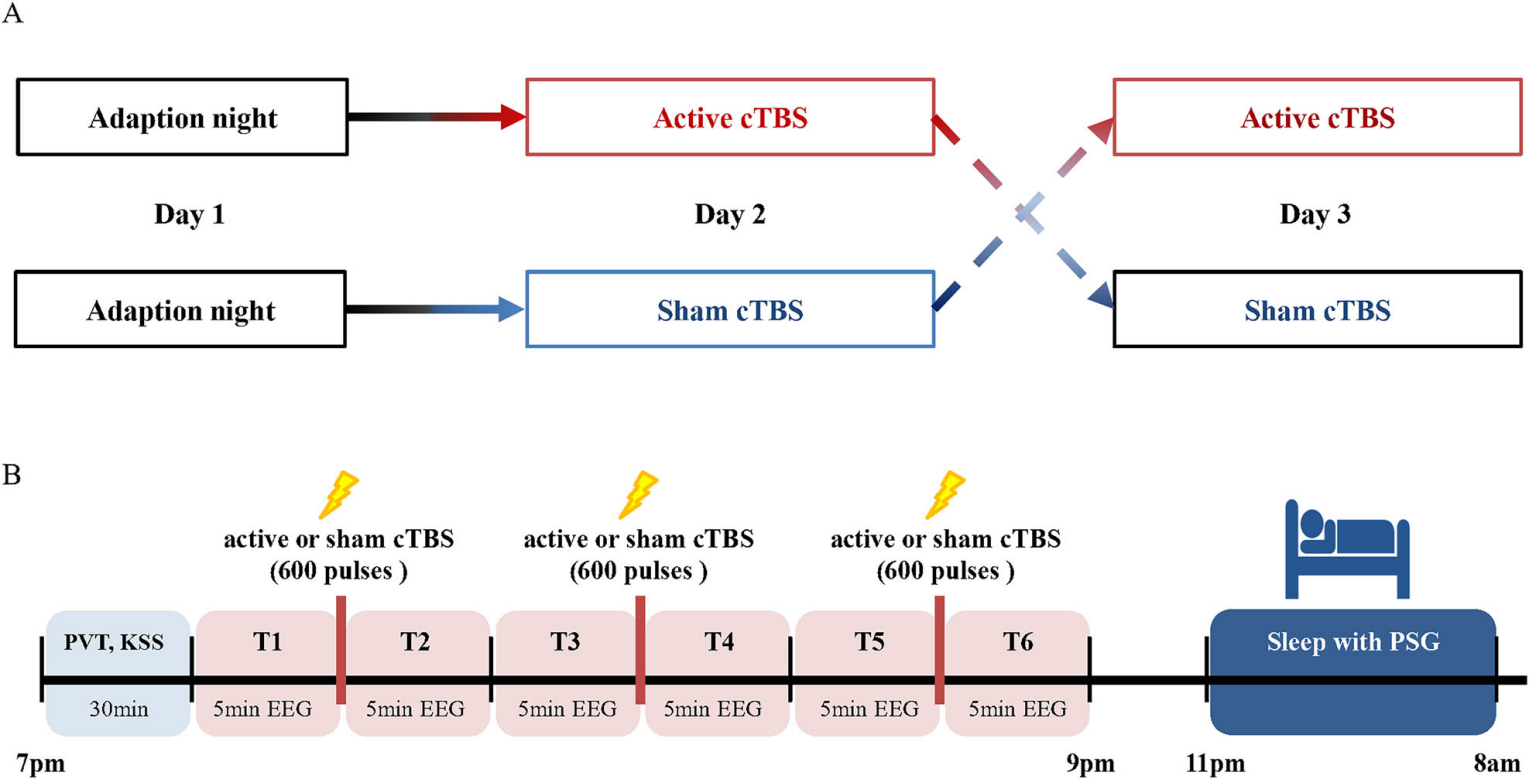
Flowchart. Participants were received either active or sham cTBS intervention in the two days in a pseudorandomized order, following an adaptation night (**A**). Participants completed the psychomotor vigilance test (PVT) and karolinska sleepiness scale (KSS) before intervention. The rsEEG was recorded for 5 min both before and after cTBS in each session. After completing three sessions of cTBS, participants were prepared for sleep with polysomnography (PSG) equipment (**B**).

The study utilized a single-blinded active-sham crossover design. During 7 days preceding study, participants maintained a regular sleep–wake schedule. Compliance was verified using sleep diaries which was described in Supplementary Materials. Participants were requested to abstain from all caffeine- and alcohol-containing beverages and from intense physical activity for 7 days preceding study. The entire experimental protocol lasted 3 nights, including 1 night for adaptation to the lab environment and screening other sleep disorders, and the 2 nights for active or sham cTBS intervention (Fig. 1A). The order of active and sham intervention was pseudorandomized using a computer-generated list. For each night, participants arrived at the laboratory 4 h before their habitual sleep time, and completed the psychomotor vigilance test (PVT) and Karolinska sleepiness scale (KSS) which reflect the level of vigilance and sleepiness. The laboratory illumination was maintained at 300 lux, except during sleep periods when the room was kept in complete darkness. The laboratory was a soundproof room with a temperature controlled at 23 °C.

One session of cTBS consisted of 600 pulses at 80% resting motor threshold (RMT) over right dlPFC (F4, 10–20 system), which included 3 pulses at 50 Hz repeated at a frequency of 5 Hz [16]. The right-side RMT was the lowest stimulus intensity at which TMS evokes a MEP in at least 5 out of 10 trials, with the coil positioned over the right motor cortex [19]. Both active and sham cTBS intervention comprised three sessions with a 15-min interval between each session following a prior study (Fig. 1B) [20]. The stimulation setup consisted of a Magstim Rapid magnetic stimulator (Magstim, Whitland, Dyfed, UK) and a figure-of-eight TMS coil (70 mm standard coil, Magstim Co., Whitland, Dyfed, UK). Active intervention used the coil that placed tangentially to the scalp with the handle pointing backward and 45° away from the midline. Sham intervention utilized the coil perpendicular to the right dlPFC to provide auditory stimulation and thereby improve blinding.

Eyes-closed rsEEG was recorded in six timepoints which included *pre* and *post* of the first session cTBS (T1 and T2), the second session cTBS (T3 and T4), and the third session cTBS (T5 and T6). During rsEEG recordings, participants were instructed to keep their eyes closed, stay relaxed and avoid falling asleep. After three sessions cTBS, patients were outfitted with a polysomnography monitoring device and instructed to retire to bed at their habitual bedtime. After full night’s sleep, patients filled out a brief questionnaire which is described in Supplementary Materials.

### EEG recording and processing

EEG was recorded by the amplifier (BrainAmp DC EEG acquisition system of Brain Products GmbH, Gilching, Germany) with 64 channels. Impedance per electrode was maintained below 5 kΩ, and online bandpass filtering parameter was 0.5–100 Hz, along with a sampling rate of 1000 Hz. EEG data were preprocessed following the standard procedures, which included the common averaging-referencing, 1–60 Hz bandpass filtering, 50 Hz notch filtering, 5-s-length data segmentation, and artifact removal (±100 μV as threshold). Additionally, as denser electrodes might provoke more severe volume conduction effects on connectivity, sparse electrodes were used to reduce the effect of volume conduction on EEG networks [21]. Concretely, 21 of 64 channels were selected in our present study to perform the analyses described below (Fp1, Fpz, Fp2, F3, Fz, F4, C3, CZ, C4, P3, Pz, P4, O1, Oz, O2, F7, F8, T7, T8, P7, and P8).

### Power spectral density

Power spectral density (PSD) of rsEEG was calculated using the Welch’s averaged modified periodogram method on artifact-free consecutive, nonoverlapping 5 s epochs (Hamming window), in typical frequency bands, including delta (1–4 Hz), theta (5–8 Hz), alpha (8–13 Hz), beta (13–30 Hz), and gamma (30–60 Hz). First, to detect PSD in baseline between active and sham intervention, PSD of all five frequency bands in T1 was compared between two interventions. Second, to explore the cumulative effect of three sessions cTBS, paired two-tailed *t*-tests was used to compare changes of PSD in the baseline and *post*-cTBS of three sessions (i.e., T1 vs. T2, T1 vs. T4, T1 vs. T6). Third, paired two-tailed *t*-tests were conducted to compare the PSD in *pre-* and *post*- cTBS of each session (i.e., T1 vs. T2, T3 vs. T4, T5 vs. T6). The threshold for statistical significance was set at *p* < 0.05 after false discovery rate (FDR) correction.

### Weighted network

When constructing the brain networks for baseline and *post*-cTBS, the synchronization likelihood between pairwise electrodes was considered [22]. As suggested previously, phase locking value (PLV) was utilized in estimating the phase synchronization among pairwise signals [23]. Furthermore, when exploring the differences between baseline and *post*-cTBS, analyses were performed within typical frequency bands as well as above. Specifically, we set 21 electrodes as the network nodes, and estimated interelectrode interactions were then regarded as the network edges. PLV was used to calculate the phase-synchronization between pairwise electrodes, which gave an adjacency matrix with dimensions of 21 × 21 [24]. Furthermore, the PLV network differences between baseline and *post*-cTBS were also explored within each typical frequency band by paired two-tailed *t*-tests with FDR correction. To explore the cumulative effect of cTBS, the PLV of all five frequency bands was compared between baseline and *post*-cTBS using paired two-tailed *t*-tests. The threshold for statistical significance was set at *p* < 0.05 after FDR correction.

### Network property

To further quantify the degree of phase synchronization, network properties including the clustering coefficient (CC) and characteristic path length (CPL), global efficiency (GE), and local efficiency (LE) were calculated based on the constructed PLV network using the brain connectivity toolbox (http://www.nitrc.org/projects/bct/) [25]. As proved, CC is a measure of the degree to which nodes in a network tend to cluster together. A high CC thereby indicates that nodes in a network tend to be highly interconnected [26]. A low CPL indicates that information can be transmitted quickly and efficiently across the network, and vice versa [27]. GE and LE are measures of the efficiency of information transfer in a network at the global and local levels, respectively [27]. Together, GE and LE provide complementary information about the efficiency of information transfer in a network at different scales, and can be used to assess the impact of different types of perturbations or interventions on network function. Here, these properties were calculated from the weighted EEG networks without any thresholding processing. Two-way repeated measures analysis of variance (ANOVA) was then adopted to determine whether there was a statistically significant interaction effect between two interventions across time. Then, the network properties of each timepoint were compared between two interventions using a paired *t*-test with FDR correction.

### PSG recording and processing

Standard PSG was performed on the experimental PSG night by using a digital polysomnographic monitor (Greal Series, Compumedics, Victory, Australia). The following biological variables were monitored continuously: 6-channel EEG (F4-M1, C4-M1, O2-M1, F3-M2, C3-M2 and O1-M2), electrooculography, electromyography, electrocardiography, inductance plethysmography belts, pulse oximetry, thermistor and nasal pressure transducers. Data was scored manually by a sleep specialist according to the AASM scoring system [28]. Sampling rate of each system was 512 Hz, and the impedance was maintained below 5 kΩ per electrode. Last 15 participants were recorded with 19 channel EEG derivations described in the method of Supplementary Materials. PSG data were scored by one independent, experienced scorer who was kept blind to the type of intervention, in 30 s epochs using the criteria defined by the AASM [28]. The sleep stage characteristics, including time in bed (TIB), total sleep time (TST), sleep efficiency (SE), sleep latency (SL), and wake after sleep onset (WASO), NREM, and REM, were compared between the two interventions of subjects using paired *t*-tests with FDR correction. Additionally, the NREM–REM sleep cycles were determined on manually scored sleep stages according to the criteria of Feinberg and Floyd [29].

The preprocessing procedures were then applied, which included averaging-referencing, 1–60 Hz bandpass filtering, 50 Hz notch filtering, and 30-s-length data segmentation. Portions in which the absolute amplitude of the EEG signal exceeded 100 μV were marked as artifacts. Channels containing artifacts for more than 50% of the recording time were excluded from the analysis.

### Power spectral density and weighted network

The PSD and PLV analysis of sleep PSG, as above, was used to calculate the spectral power and phase synchronization during the NREM of the first sleep cycle. Both rTMS and cTBS locally enhanced synaptic plasticity in the stimulated cortical regions [30, 31]. The F4 electrode, a common target for rTMS treatment of insomnia disorders, reflects the modulation effects of TMS- and cTBS-induced slow oscillations through its power changes. To explore the effects of cTBS during wakefulness to subsequent sleep, the change of PSD values at the F4 electrode was calculated by subtracting the post-intervention PSD values from the baseline values (ΔPSD = PSD_T6_−PSD_T1_). Z-scores of ΔPSD were computed using means and standard deviations generated from each band. We conducted a correlation analysis between the ΔPSD during wakefulness, and the PSD during the NREM of the first sleep cycle.

## Results

Forty-one right-handed patients with insomnia disorder (13 males; age: 40.63 ± 12.94 years) were included in this study (Table S1). Levels of vigilance and sleepiness before intervention were not significantly different between two experimental days based on PVT median reaction time and KSS score (Table S2). PSD of five frequency bands was not significant different between two interventions in baseline.

### Increased spectral power of delta and theta bands with cumulative effects

**Fig. 2:**
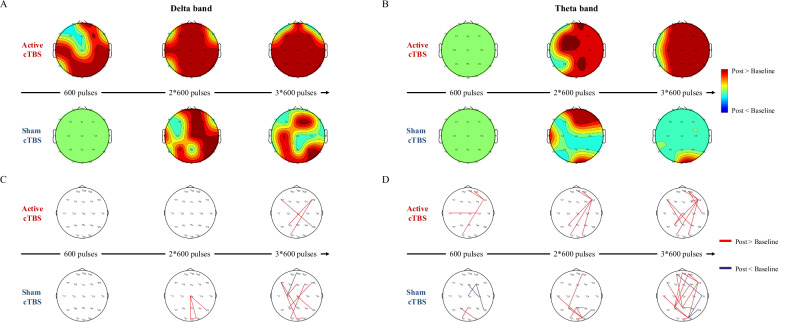
Increased spectral power and phase locking value of the delta and theta bands. The spectral power of delta (**A**) and theta (**B**) bands, as well as the phase locking value (PLV) network of delta (**C**) and theta (**D**) bands, were compared between post-cTBS and baseline.

Compared PSD in baseline (T1) with *post*-cTBS of each session (T2/4/6), PSD of delta and theta increased from posterior to global brain area with a cumulative effect as active session proceeded (*p*_*FDR*_ < 0.05; Fig. 2A and B). However, PSD of delta and theta bands was increased locally after sham session (*p*_*FDR*_ < 0.05; Fig. 2A and B). Interestingly, for gamma band, a widespread global increasing of PSD was observed across electrode sites Fp2, F4, P3, P8, O2, and Oz after first cTBS session (*p*_*FDR*_ < 0.05, Fig. S3). With administration of second cTBS session, this effect became more localized specifically to stimulated frontal cortex at electrode site F4. However, the effect did not reach statistical significance following the third cTBS session (*p*_*FDR*_ > 0.05, Fig. S3). Only in sham interventions not active interventions, PSD of the alpha band exhibited a significant decrease over three sessions (Fig. S1). The PSD of the beta band was decreased significantly between occipital and parietal regions in both interventions (Fig. S2).

PSD of these bands between *pre-* and *post*-cTBS of each session is displayed in Supplementary Materials (Fig. S5). PSD of delta band exhibited an increase solely when comparing the *pre-* and *post*-cTBS of first active session, and no changes in all sham sessions. PSD of alpha band showed an increase in second active session and a decrease in first sham session (Fig. S5C). PSD of gamma band increased globally (Fp2, F4, P3, P8, O2, and Oz) after first active session, and increased locally in electrode site F4 after second active session (Fig. S5E). PSD of theta and beta band exhibited no changes between *pre-* and *post*-cTBS of all active and sham sessions.

### Increased phase synchronization between frontal-occipital networks in delta and theta bands

Comparing between baseline and *post*-cTBS of each session, the PLV network of delta and theta bands have similar edges between frontal-occipital regions (*p*_*FDR*_ < 0.005; Fig. 2C and D). In PLV network of delta band, edges between the right frontal cortex and parietal/occipital cortex were enhanced following third session active cTBS (F8-P3 and F8-O1; Fig. 2C). After sham cTBS, edges between frontal cortex and parietal/occipital cortex were also enhanced (Fig. 2C).

In PLV network of theta band, edges between the right frontal cortex and parietal/occipital cortex (F8-Fpz, F8-Fp2 and F8-O1; Fig. 2D) were enhanced following first session active cTBS and remained consistently enhanced after the subsequent two sessions. In addition, with the number of cTBS sessions increased, the number of edges with significant changes grew. After second and third session active cTBS, the right frontal cortex electrode sites at F8 functioned as a rich hub, displaying heightened connectivity with other nodes, notably within the frontal area (F8-Fp2 and F8-Fpz), as well as the parietal/occipital areas (F8-P3, F8-O1, and F8-O2). Following the second and third session of sham cTBS, edges between the parietal cortex and occipital cortex (P3-O2, Pz-P4, Oz-O2, T7-O2; Fig. 2D) were consistently enhanced.

### Higher communication between frontal-occipital networks of delta and theta bands

**Fig. 3:**
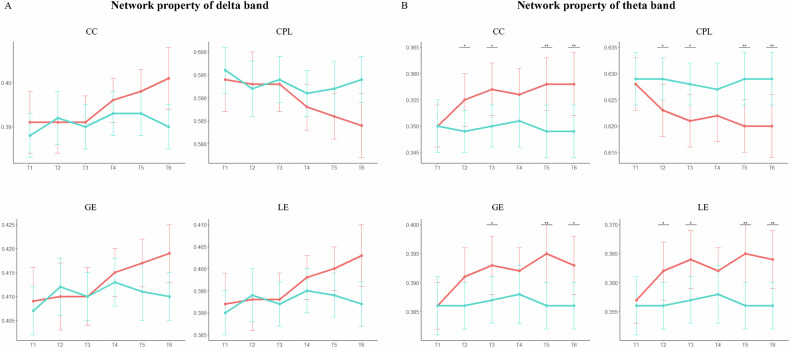
The identified differences in network properties of delta and theta bands. The two-way repeated ANOVA results of network properties were insignificant between active (red line) and sham (blue line) interventions in the delta band (**A**) and theta band (**B**). The post *t*-test of two intervention were significantly different at each timepoint. *p_*FDR*_ < 0.05; **p_*FDR*_ < 0.01. CC clustering coefficient, CPL characteristic path length, GE global efficiency, LE local efficiency.

For both active and sham cTBS interventions, the network properties, comprising CC, GE, LE, and CPL, were calculated and then compared between *pre*- and *post*-cTBS through statistical analysis. As displayed in Fig. 3, during the cTBS session, the CC, GE, and LE showed an upward trend in the delta (Fig. 3A) and theta bands (Fig. 3B), and the CPL showed a downward trend in active intervention. However, the four network properties had no consistent trend after the sham intervention. Two-way repeated ANOVA indicated that no significant interaction effect was observed between six time points and interventions for both delta and theta networks in terms of network properties. Following this, paired *t*-test with FDR correction was utilized to compare the network properties between the two interventions, at each timepoint. As exhibited, in compared with the sham stimulation, the CC and LE of the delta band were marginally significantly stronger in T6 after active intervention (CC: *p* = 0.073; LE: *p* = 0.084; Fig. 3A, Table S5). Notably, the network efficiency (CC, GE, LE) of the theta band in active interventions was significantly higher than that after the sham intervention, especially in T2, T3, T5 and T6 (*p*_*FDR*_ < 0.05, Fig. 3B, Table S5). The other bands were not significant in these network properties between two interventions (Fig. S4, Table S6).

### The spectral power of the delta and theta bands in subsequent sleep

**Fig. 4:**
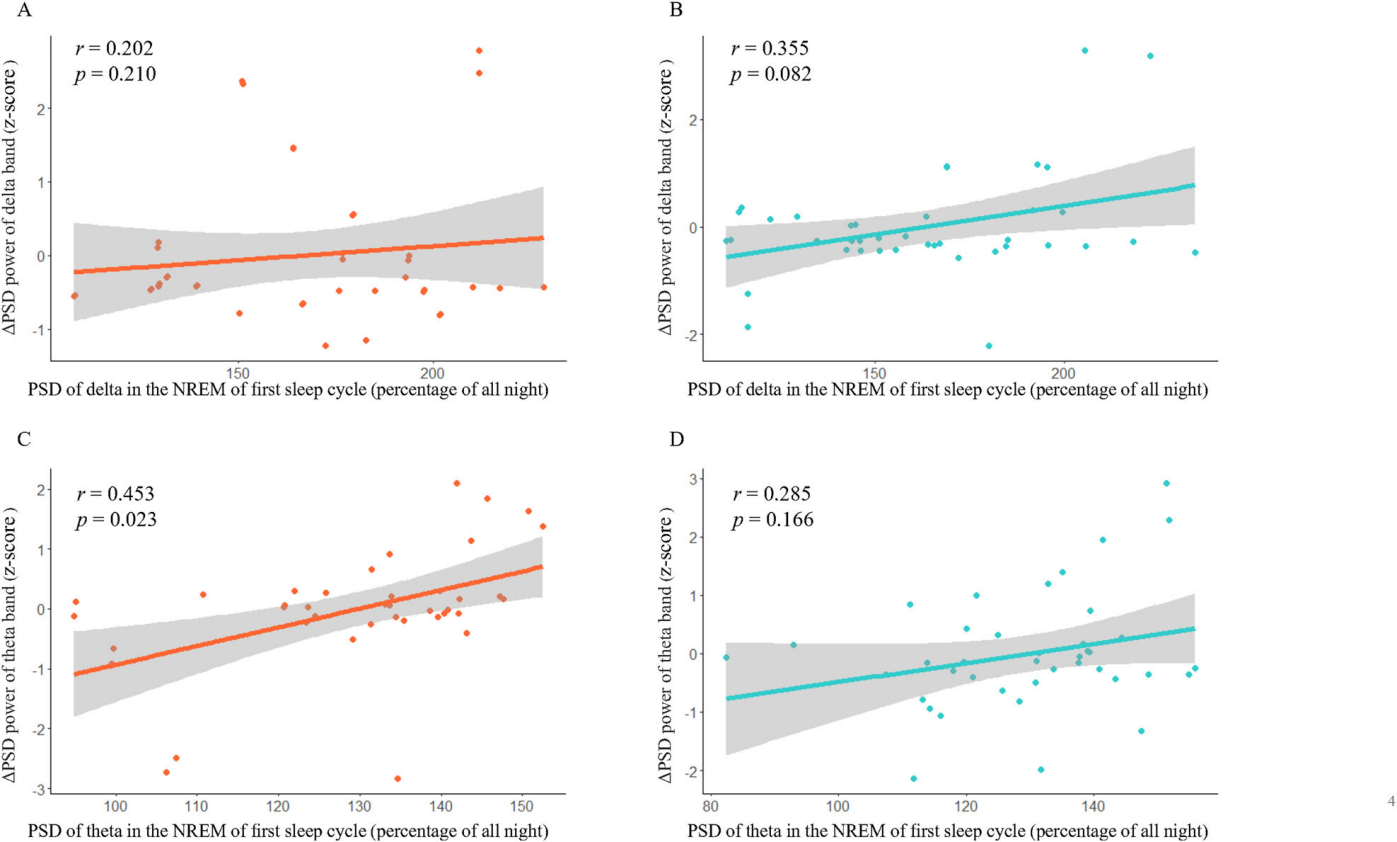
The relationship between spectral power after intervention during wakefulness and subsequent sleep onset. The ΔPSD of delta band (ΔPSD = PSD_T6_ - PSD_T1_) at the F4 electrode was not significantly related with that during the NREM of the first sleep cycle in both active (**A**) and sham intervention (**B**). The ΔPSD of theta band was significantly related with the spectral power of theta band during the NREM of the first sleep cycle in active intervention (**C**), and was not significantly in sham intervention (**D**).

A correlation analysis was conducted to explore the relationship between Z-scores of ΔPSD during wakefulness and the PSD during subsequent sleep. In both active and sham intervention, the ΔPSD of delta band was not significantly related with that during the NREM of the first sleep cycle (Fig. 4A and B). However, the ΔPSD of theta band in active intervention was significantly related with the spectral power of theta band during the NREM of the first sleep cycle (*r* = 0.453, *p* = 0.023; Fig. 4C and D).

### The objective and subjective sleep quality during subsequent sleep

Paired *t*-test analysis was conducted to examine the difference of sleep stages after active and sham cTBS interventions. The results revealed that duration of NREM sleep was longer following active cTBS (312.68 ± 45.87 min) than following sham intervention (299 ± 49.79 min, *p* = 0.037, Table S3). However, the difference did not reach statistical significance after FDR correction (*p*_*FDR*_ = 0.396, Table S3). Additionally, results of the subjective sleep diary suggested that subjective TST, subjective sleep quality and restorative quality of sleep were higher in night after active intervention than after sham intervention (*p* = 0.021, Table S4). Additionally, we found that PSD and PLV of delta and theta band was enhanced in frontal-occipital networks during the NREM of first sleep cycle after active stimulation among last fifteen participants with 19-channel PSG (*p*_*FDR*_ < 0.05, Fig. S6). However, when comparing the active and sham interventions during the NREM of first sleep cycle, there were no significant differences observed in the PSD and PLV of delta and theta bands among all forty-one participants in 6-channel channels.

## Discussion

These findings indicated that spectral power of the delta and theta bands was increased after active cTBS intervention among patients with insomnia, accompanied by enhanced PLV connections between stimulated frontal areas and occipital areas. More importantly, both spectral power and PLV connections of delta and theta bands exhibited a cumulative effect with increasing pulses of cTBS. However, gamma band was increased locally and then disappeared after the last session. In addition, increased theta band during wakefulness also improved that in subsequent sleep after active intervention. cTBS modulated the spectral power and phase synchronization of low frequency oscillations within frontal-occipital network, which also modulated subsequent sleep.

TBS has been shown to modulate brain activity by mimicking theta-gamma coupling which is important in cognitive processing [32–34], while little attention has been given to exploring its influence on the spontaneous brain oscillations. Previous studies found that cTBS suppressed cortical excitability measured by motor-evoked potentials. To assess brain oscillations modulated by cTBS, the rsEEG provides a window into spontaneous cortical oscillations. In this regard, we found that the spectral power of theta band was significantly increased after active cTBS, possibly attributed to the local entrainment of brain oscillations induced by theta frequency stimulation [35]. Following all three sessions, the spectral power of theta band exhibited a cumulative effect with a global enhancement. Additionally, theta activity has been recognized as a prominent indicator of sleep need in wake EEG recordings [36, 37]. The increased delta and theta power after stimulation may promote subjective sleepiness and sleep drive [17, 38]. Moreover, our study found that alpha power was decreased during eyes-closed in sham interventions, while alpha activity is enhanced during the relaxation with closed eyes in health people. Mental stress also induced decreased alpha power which maybe indicated cortical hyperarousal of insomnia [39]. Conversely, spectral power of gamma band exhibited localized enhancement in stimulated area, which may be induced by TBS paradigm that involves administering TMS in three-pulse 50 Hz bursts. However, this enhanced gamma power subsequently diminished, which may indicate that active cTBS suppresses cortical excitability and primarily induces low-frequency rather than high-frequency activity. And the future research should further explore the spectral power of gamma band after cTBS intervention. Previous studies have categorized the effects of TMS as either inhibitory or excitatory. However, this dichotomous effect is not entirely accurate and has certain limitations in practical application [40]. We have also observed substantial individual differences in the responses to cTBS in other experiments and clinical applications [41]. We propose that these individual differences are likely related to the brain state, synaptic plasticity, circadian rhythm, and the parameters of cTBS intervention [42]. Future research should further investigate individual differences in the response to cTBS interventions.

Our study also revealed that multiple sessions cTBS produce a cumulative increasing in spectral power of delta and theta bands. A previous study revealed that TMS induced an increasing of corticospinal excitability across multiple stimulation blocks [43]. One session of cTBS with 600 pulses induced local changes in EEG activity. Following the second session of cTBS, spectral power of delta and theta activities exhibited an upward trend, which further strengthened after the third session. All three sessions cTBS induced and enhanced endogenous brain oscillations in low frequency band. Moreover, PLV network after stimulation showed a higher efficiency of local and global information communication across the entire network, which also exhibited a cumulative increasing trend. Long-range neuronal synchronization was improved, especially in delta and theta band, which may be guided through underlying brain architecture [31]. We observed that cTBS not only induced local oscillations but also modulated global brain oscillations through the modulation of phase synchronization between low-frequency oscillations [44]. Synchronous brain activity in the brain is mediated by structural white matter pathways and is highly dependent on topological brain properties [45].

Through delivering TMS pulses at specific frequencies to targeted brain regions, both rTMS and cTBS modulate cortical activity within the target area, and subsequently influence the activity in other interconnected brain regions [46, 47]. As revealed by PLV network, we found that F8 electrode was located in close to the stimulated F4 electrode site, and displayed strengthened phase synchronization with the parietal and occipital areas, which provides insight into how local stimulation can influence global brain activity. Most often, simple projections are used between the coil position at the scalp and the assumed direct cortical stimulation region. However, the peak of the TMS-induced electric field is not always located directly underneath the stimulation coil [48]. TMS-evoked activity also propagates throughout the within modules of the stimulated network rather than the targeted region [49]. Many studies have demonstrated the propagation of rTMS effects via structural connections near the stimulated area to other brain structures [48, 50]. Direct electrical TBS induces theta oscillations through simultaneous intracranial EEG in neurosurgical epilepsy patients [31]. Previous studies have demonstrated that insomnia appears to alter the functional connectivity between the frontal, parietal, and occipital cortices [51, 52]. Specifically, patients with primary insomnia exhibit decreased connectivity within regions of the right frontoparietal network, including the superior parietal cortex and the superior frontal cortex [53, 54]. Both strengthened PLV connection after stimulation, along with the increased efficiency of local and global information communication, provided evidences about the induction and stabilization of brain oscillations within the frontal-occipital network by cTBS. Given the comparison of changes in the PLV matrix (21 × 21 electrodes) before and after stimulation, significant variations were also observed in the connections following sham stimulation. Future research could further investigate the changes in PLV connectivity between the targeted brain regions and other brain regions by establishing regions of interest.

To improve sleep quality, it is important to determine whether the modulation of low-frequency oscillation by cTBS during wakefulness is sustained for sleep. cTBS lead to inhibitory aftereffects lasting from minutes to hours during wakefulness [55]. Although a recent study reported that cTBS during wakefulness improved objective sleep duration and sleep efficiency during subsequent sleep [18]. Our study found that cTBS was not effective in improving objective sleep latency or total sleep time measured by PSG during the subsequent night. But increased theta power during wakefulness induced increased spectral power of theta band during sleep onset, which indicated cTBS could influence subsequent sleep by modulation spectral power. In addition, global topological distribution of delta and theta band during sleep onset was similar with changes after cTBS during wakefulness [56, 57]. And the changes were only significant in measures with 19-channel PSG than 6-channel PSG. The high-density PSG was necessary in future study about modulation of stimulation in sleep. The process of sleep-wake transitions involves the synergistic actions of multiple nerve nuclei and neurotransmitters [58]. Neurophysiological evidence reveals that orexinergic neurons depolarize prior to the onset of EEG activation during transitions from sleep to wakefulness. Suvorexant, an orexin receptor antagonist, has been shown to improve both subjective and objective sleep quality without major changes in cortico-thalamic neurophysiology [59]. However, the potential neural mechanisms underlying cTBS-induced aftereffects require further exploration using additional measurements beyond EEG.

Additionally, this study is limited by its crossover design with 1-day apart between interventions. Notably, the duration may prove to be insufficient for certain changes induced by cTBS to fully recover. Thus, interference of cTBS interventions as well as an impact on sleep quality cannot be excluded. Another limitation is that sham cTBS was gentler than active cTBS, which might have attenuated for detection of small effects. And sham cTBS coil was perpendicular to the right dlPFC, which is unlikely given the similar muscle twitching and pain of the active condition. Overall, present study provides novel insights into underlying mechanisms of the modulatory effects of cTBS on brain oscillations. The cTBS delivered over multiple sessions can produce cumulative lasting changes and improve the sleep after stimulation, which has shown potential utility as a therapeutic intervention for insomnia patients.

## Conclusion

The study demonstrated that cTBS intervention in insomnia patients selectively increased delta and theta band spectral power, and enhanced PLV connectivity between frontal and occipital areas. This modulation also extended to influence theta power during subsequent sleep onset period. Collectively, these findings provide a robust theoretical foundation for further investigating the therapeutic potential of long-term cTBS in the treatment of insomnia disorders.

## Supplementary information

SUPPLEMENTAL Tables and Figures

## Data Availability

The data that support the findings of this study are available on request from the corresponding author. The data are not publicly available because they contain information that could compromise the privacy of research participants.
