# Supplementary material for: Modulation of brain oscillations by continuous theta burst stimulation in patients with insomnia
Source: Transl Psychiatry. 2025 Oct 17;15:416. doi: 10.1038/s41398-025-03605-y (PMC12534500; doi:10.1038/s41398-025-03605-y)
Supplement: Supplementary file 1 — SUPPLEMENTAL Tables and Figures [file 41398_2025_3605_MOESM1_ESM.docx]

**METHODS**

**Sleep diary**

The sleep diary queries (1) time into bed, (2) time of sleep attempt, (3) time it took to fall asleep, (4) number of awakenings during the main sleep period, (5) total estimated duration of this awakenings, (6) rise time, (7) total self-reported sleep time, (8) subjective report of sleep quality, and (9) how would you rate the restorative quality of your sleep (a 6-point Likert-type item with a very simple answer format: 0 = nonrestorative and 5 = very restorative). Of these nine items, the first seven are used to derive estimates of other sleep indices. Sleep latency (SL) is simply the recorded time to fall asleep (item 3). Similarly, wakefulness after sleep onset (WASO) is equivalent to the total duration of the nighttime awakenings (item 5). Time in bed (TIB) is calculated by examining the difference between time into bed and rise time (items 1 and 6). Total sleep time (TST) equivalent to the total duration of the total sleep time (item 7). Finally, sleep efficiency (SE) is calculated by dividing TST by TIB (i.e., SE is equivalent to the amount of time spent sleeping compared to the total amount of time the individual spent in bed).

**PSG**

Standard polysomnography (PSG) was performed on the experimental PSG night by using a digital polysomnographic monitor (Greal Series, Compumedics, Victory, Australia). The last 15 participants were recorded with 19-channel EEG (Fp1-M2, Fp2-M1, Fz-M2, F3, F4-M1, F7-M2, F8-M1, Cz-M2, C3-M2, C4-M1, Pz-M2, P3, P4-M1, O1-M2, O2-M1, T3-M2, T4-M1, T5, and T6-M1), electrooculography, submental and anterior tibial electromyography, and electrocardiography. Inductance plethysmography belts were used to monitor chest and abdominal movements. Thermistor and nasal pressure transducers were used to monitor airflow. Snoring was recorded using a microphone. Pulse oximetry was used to measure arterial oxygen saturation (SpO2).

**RESULTS**

**Table S1. Demographic Information**

|  |  | | Insomnia patients (N=41) | |
| --- | --- | --- | --- | --- |
| Age |  | | | 40.63±12.94 |
| Sex | Male | | | 13 |
|  | Female | | | 28 |
| Education, years | | | | 16.20±2.98 |
| Insomnia duration, months | | | | 55.23±73.55 |
| ISI score | |  | | 16.63±3.92 |
| PSQI score | | | | 12.46±3.01 |
| PHQ-9 score | |  | | 8.45±4.65 |
| GAD-7 score | |  | | 7.08±5.40 |
| ESS score | |  | | 7.41±4.35 |
| MoCa score | |  | | 26.7±2.92 |

**Table S2. The PVT median reaction time and KSS score before stimulation.**

|  | active cTBS | sham cTBS | *p* |
| --- | --- | --- | --- |
| PVT median reaction time (min) | 334.85±32.42 | 337.93±33.59 | 0.370 |
| PVT lapse | 4.83±5.33 | 4.62±3.98 | 0.773 |
| KSS score | 2.96± 1.08 | 2.85±0.88 | 0.755 |

**Table S3. Objective sleep stage for overnight sleep after stimulation**

|  | active cTBS | sham cTBS | *p* | *p_FDR_* |
| --- | --- | --- | --- | --- |
| TIB (min) | 464.34±53.68 | 458.02±50.31 | 0.350 | 0.550 |
| TST (min) | 392.46±60.1 | 379.41±63.18 | 0.118 | 0.431 |
| SE (%) | 92.53±10 | 92.26±13 | 0.755 | 0.878 |
| SL (min) | 29.32±9.82 | 30.15±10.08 | 0.620 | 0.853 |
| WASO (min) | 36.05±33.09 | 43.88±48.05 | 0.220 | 0.431 |
| NREM (min) | 312.68±45.87 | 299±49.79 | 0.037* | 0.396 |
| REM (min | 79.8±26.01 | 80.44±24.13 | 0.867 | 0.878 |
| NREM 1 (%) | 8.34±2.87 | 8.9±4.49 | 0.233 | 0.431 |
| NREM 2 (%) | 55.07±11.02 | 53.37±10.37 | 0.072 | 0.396 |
| NREM 3 (%) | 16.61±9.81 | 16.71±9.28 | 0.878 | 0.878 |
| REM (%) | 20.12±4.96 | 21.05±5.25 | 0.235 | 0.431 |

**Table S4. Subjective sleep diary for overnight sleep after stimulation**

|  | Active cTBS | Sham cTBS | *p* |
| --- | --- | --- | --- |
| TIB (min) | 437.56±56.52 | 439.39±60.24 | 0.925 |
| TST (min) | 370.74±66.83 | 336.93±70.01 | 0.021 |
| SE (%) | 85.46±14.49 | 77.18±14.25 | 0.021 |
| SL (min) | 25.76±20.91 | 26.10±13.30 | 0.925 |
| WASO (min) | 37.05±36.53 | 46.49±55.34 | 0.331 |
| Sleep quality | 3.29±1.05 | 2.66±0.91 | 0.043 |
| Restorative quality of sleep | 3.27±1.00 | 2.68±0.91 | 0.042 |

**Table S5. The network properties of the delta and theta band**

|  | | delta band | | |  | | | theta band | | |
| --- | --- | --- | --- | --- | --- | --- | --- | --- | --- | --- |
|  | active cTBS | | sham cTBS | *p_FDR_* | |  | active cTBS | | sham cTBS | *p_FDR_* |
| **CC** |  | |  |  | |  |  | |  |  |
| T1 | 0.391+0.043 | | 0.388+0.034 | 0.615 | |  | 0.35+0.028 | | 0.35+0.033 | 0.813 |
| T2 | 0.391+0.044 | | 0.392+0.041 | 0.883 | |  | 0.355+0.034 | | 0.349+0.027 | 0.028 |
| T3 | 0.391+0.037 | | 0.39+0.031 | 0.801 | |  | 0.357+0.032 | | 0.35+0.025 | 0.016 |
| T4 | 0.396+0.032 | | 0.393+0.034 | 0.566 | |  | 0.356+0.029 | | 0.351+0.032 | 0.195 |
| T5 | 0.398+0.034 | | 0.393+0.035 | 0.324 | |  | 0.358+0.034 | | 0.349+0.029 | 0.006 |
| T6 | 0.401+0.042 | | 0.39+0.031 | 0.076 | |  | 0.358+0.035 | | 0.349+0.029 | 0.002 |
| **CPL** |  | |  |  | |  |  | |  |  |
| T1 | 0.594+0.044 | | 0.596+0.035 | 0.649 | |  | 0.628+0.029 | | 0.629+0.034 | 0.913 |
| T2 | 0.593+0.045 | | 0.592+0.041 | 0.889 | |  | 0.623+0.034 | | 0.629+0.028 | 0.04 |
| T3 | 0.593+0.039 | | 0.594+0.032 | 0.84 | |  | 0.621+0.032 | | 0.628+0.026 | 0.029 |
| T4 | 0.588+0.034 | | 0.591+0.035 | 0.615 | |  | 0.622+0.03 | | 0.627+0.033 | 0.175 |
| T5 | 0.586+0.035 | | 0.592+0.036 | 0.312 | |  | 0.62+0.034 | | 0.629+0.03 | 0.008 |
| T6 | 0.584+0.042 | | 0.594+0.032 | 0.092 | |  | 0.62+0.035 | | 0.629+0.03 | 0.005 |
| **GE** |  | |  |  | |  |  | |  |  |
| T1 | 0.409+0.043 | | 0.407+0.034 | 0.734 | |  | 0.386+0.028 | | 0.386+0.032 | 0.963 |
| T2 | 0.41+0.044 | | 0.412+0.04 | 0.824 | |  | 0.391+0.031 | | 0.386+0.026 | 0.057 |
| T3 | 0.41+0.038 | | 0.41+0.031 | 0.938 | |  | 0.393+0.03 | | 0.387+0.024 | 0.027 |
| T4 | 0.415+0.033 | | 0.413+0.034 | 0.686 | |  | 0.392+0.027 | | 0.388+0.03 | 0.093 |
| T5 | 0.417+0.034 | | 0.411+0.035 | 0.296 | |  | 0.395+0.031 | | 0.386+0.028 | 0.009 |
| T6 | 0.419+0.041 | | 0.41+0.031 | 0.118 | |  | 0.393+0.033 | | 0.386+0.028 | 0.013 |
| **LE** |  | |  |  | |  |  | |  |  |
| T1 | 0.392+0.043 | | 0.39+0.034 | 0.644 | |  | 0.357+0.028 | | 0.356+0.033 | 0.872 |
| T2 | 0.393+0.043 | | 0.394+0.04 | 0.868 | |  | 0.362+0.033 | | 0.356+0.027 | 0.032 |
| T3 | 0.393+0.037 | | 0.392+0.031 | 0.84 | |  | 0.364+0.031 | | 0.357+0.025 | 0.017 |
| T4 | 0.398+0.032 | | 0.395+0.033 | 0.596 | |  | 0.362+0.028 | | 0.358+0.031 | 0.155 |
| T5 | 0.4+0.033 | | 0.394+0.035 | 0.32 | |  | 0.365+0.033 | | 0.356+0.028 | 0.006 |
| T6 | 0.403+0.042 | | 0.392+0.031 | 0.084 | |  | 0.364+0.034 | | 0.356+0.028 | 0.003 |

**Table S6. The network properties of alpha, beta, and gamma band**

|  | alpha band | | | beta band | | | gamma band | | |
| --- | --- | --- | --- | --- | --- | --- | --- | --- | --- |
|  | active cTBS | sham cTBS | *p_FDR_* | active cTBS | sham cTBS | *p_FDR_* | active cTBS | sham cTBS | *p_FDR_* |
| **CC** |  |  |  |  |  |  |  |  |  |
| T1 | 0.436+0.078 | 0.441+0.075 | 0.605 | 0.321+0.049 | 0.32+0.052 | 0.98 | 0.281+0.048 | 0.265+0.032 | 0.017 |
| T2 | 0.428+0.069 | 0.417+0.068 | 0.083 | 0.319+0.044 | 0.319+0.05 | 0.945 | 0.272+0.038 | 0.27+0.033 | 0.554 |
| T3 | 0.417+0.072 | 0.409+0.064 | 0.150 | 0.313+0.042 | 0.315+0.046 | 0.757 | 0.271+0.042 | 0.271+0.035 | 0.993 |
| T4 | 0.423+0.075 | 0.406+0.062 | 0.110 | 0.323+0.046 | 0.318+0.048 | 0.34 | 0.274+0.047 | 0.267+0.031 | 0.234 |
| T5 | 0.417+0.074 | 0.416+0.067 | 0.927 | 0.316+0.042 | 0.324+0.044 | 0.161 | 0.272+0.039 | 0.272+0.032 | 0.847 |
| T6 | 0.412+0.067 | 0.408+0.071 | 0.742 | 0.321+0.049 | 0.314+0.053 | 0.184 | 0.28+0.063 | 0.264+0.037 | 0.07 |
| **CPL** |  |  |  |  |  |  |  |  |  |
| T1 | 0.543+0.079 | 0.539+0.076 | 0.616 | 0.655+0.049 | 0.654+0.052 | 0.913 | 0.697+0.047 | 0.713+0.034 | 0.014 |
| T2 | 0.551+0.069 | 0.562+0.068 | 0.077 | 0.654+0.045 | 0.655+0.051 | 0.798 | 0.705+0.04 | 0.708+0.035 | 0.532 |
| T3 | 0.562+0.072 | 0.57+0.064 | 0.152 | 0.661+0.043 | 0.659+0.047 | 0.755 | 0.707+0.044 | 0.706+0.036 | 0.837 |
| T4 | 0.555+0.074 | 0.572+0.062 | 0.112 | 0.651+0.047 | 0.656+0.049 | 0.323 | 0.704+0.047 | 0.711+0.034 | 0.217 |
| T5 | 0.562+0.073 | 0.562+0.067 | 0.961 | 0.658+0.043 | 0.65+0.045 | 0.152 | 0.706+0.041 | 0.706+0.034 | 0.937 |
| T6 | 0.568+0.067 | 0.571+0.07 | 0.789 | 0.655+0.049 | 0.661+0.054 | 0.204 | 0.698+0.062 | 0.714+0.039 | 0.066 |
| **GE** |  |  |  |  |  |  |  |  |  |
| T1 | 0.462+0.074 | 0.466+0.07 | 0.623 | 0.366+0.046 | 0.368+0.05 | 0.735 | 0.324+0.044 | 0.308+0.035 | 0.01 |
| T2 | 0.456+0.064 | 0.445+0.062 | 0.069 | 0.368+0.043 | 0.367+0.048 | 0.733 | 0.317+0.04 | 0.314+0.035 | 0.518 |
| T3 | 0.446+0.066 | 0.438+0.059 | 0.133 | 0.362+0.041 | 0.364+0.044 | 0.755 | 0.314+0.044 | 0.316+0.036 | 0.683 |
| T4 | 0.453+0.069 | 0.437+0.056 | 0.107 | 0.37+0.044 | 0.366+0.046 | 0.342 | 0.318+0.046 | 0.311+0.035 | 0.188 |
| T5 | 0.446+0.067 | 0.446+0.061 | 0.933 | 0.364+0.041 | 0.372+0.043 | 0.167 | 0.316+0.041 | 0.316+0.035 | 0.974 |
| T6 | 0.44+0.063 | 0.438+0.064 | 0.854 | 0.366+0.046 | 0.361+0.052 | 0.262 | 0.322+0.058 | 0.307+0.04 | 0.065 |
| **LE** |  |  |  |  |  |  |  |  |  |
| T1 | 0.44+0.076 | 0.444+0.073 | 0.611 | 0.33+0.048 | 0.331+0.051 | 0.939 | 0.29+0.046 | 0.274+0.032 | 0.015 |
| T2 | 0.432+0.067 | 0.421+0.066 | 0.080 | 0.33+0.043 | 0.33+0.049 | 0.887 | 0.282+0.038 | 0.28+0.033 | 0.552 |
| T3 | 0.422+0.07 | 0.413+0.062 | 0.141 | 0.324+0.041 | 0.326+0.045 | 0.764 | 0.28+0.042 | 0.281+0.035 | 0.915 |
| T4 | 0.428+0.073 | 0.411+0.06 | 0.107 | 0.333+0.045 | 0.329+0.047 | 0.335 | 0.284+0.046 | 0.277+0.032 | 0.219 |
| T5 | 0.422+0.072 | 0.421+0.064 | 0.923 | 0.327+0.041 | 0.335+0.043 | 0.163 | 0.282+0.039 | 0.281+0.033 | 0.877 |
| T6 | 0.416+0.065 | 0.413+0.069 | 0.767 | 0.33+0.048 | 0.324+0.052 | 0.197 | 0.289+0.061 | 0.273+0.038 | 0.068 |

**Fig. S1. The power spectral density and phase locking value of the alpha band.**

**
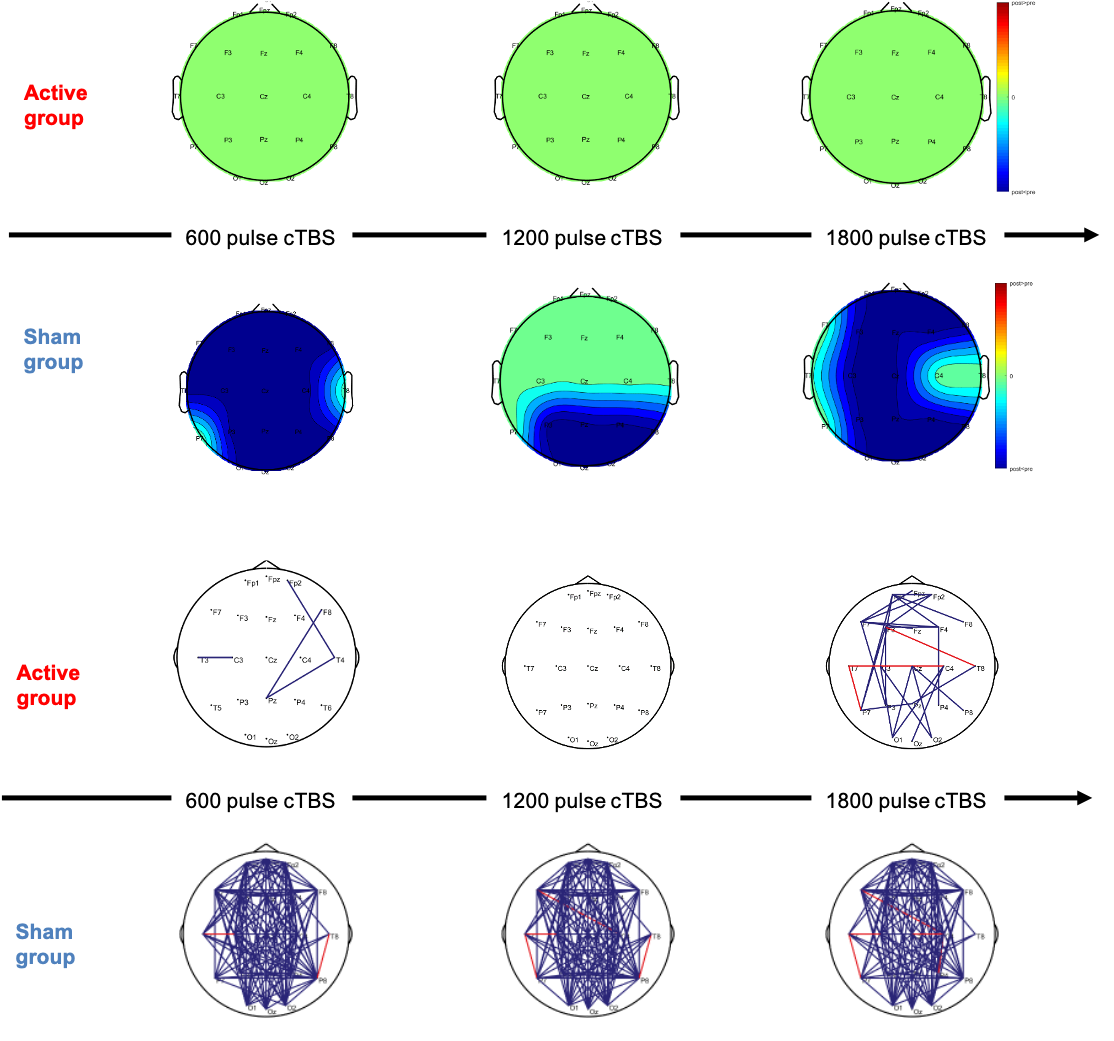
**The topological distribution of power spectral density (PSD) of the alpha band was compared to baseline (T1) in each session after active/sham cTBS. The resting network topological differences between baseline and post-cTBS in the alpha band. The red line indicated that PLV connectivity in *post*-cTBS was higher than that in baseline, and the blue lines represent that PLV connectivity in *post*-cTBS was lower than that in baseline (*p_FDR_* <0.05).

**Fig. S2. The power spectral density and phase locking value of the beta band.**


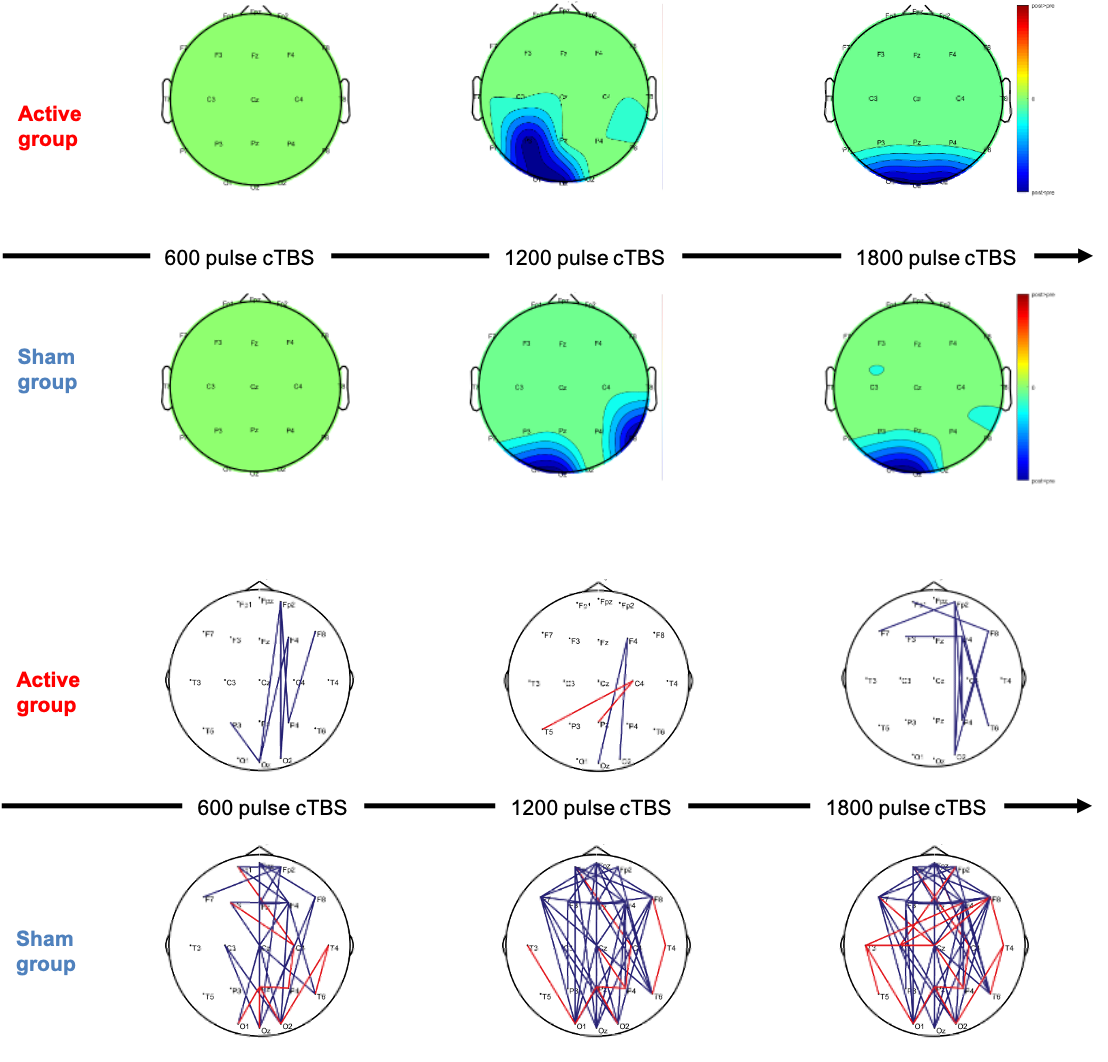


The topological distribution of power spectral density (PSD) of the beta band was compared to baseline (T1) in each session after active/sham cTBS. The resting network topological differences between baseline and *post*-cTBS in the beta band.

**Fig. S3. The power spectral density and phase locking value of the gamma band.**


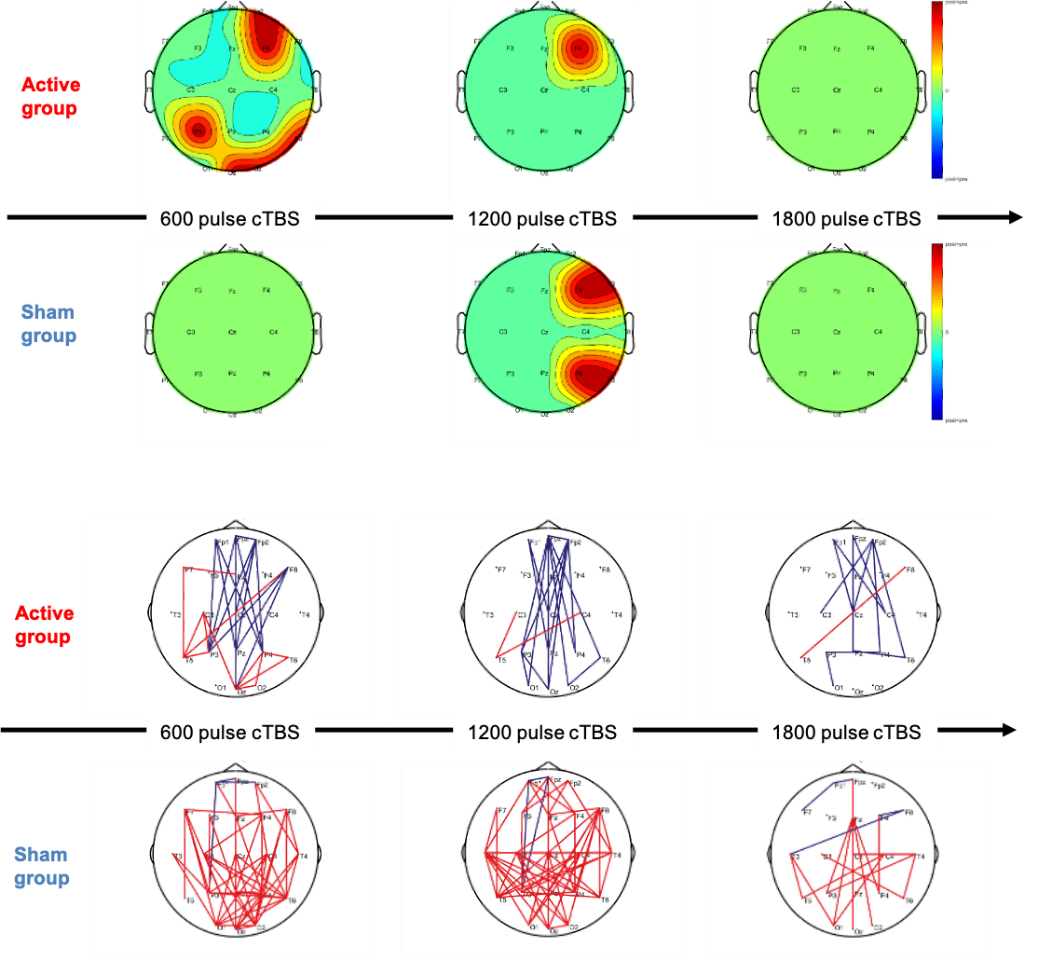


The topological distribution of power spectral density (PSD) of the gamma band was compared to baseline (T1) in each session after active/sham cTBS. The resting network topological differences between baseline and *post*-cTBS in the gamma band.

**Fig. S4. The differences in network properties of alpha, beta, and gamma bands.**


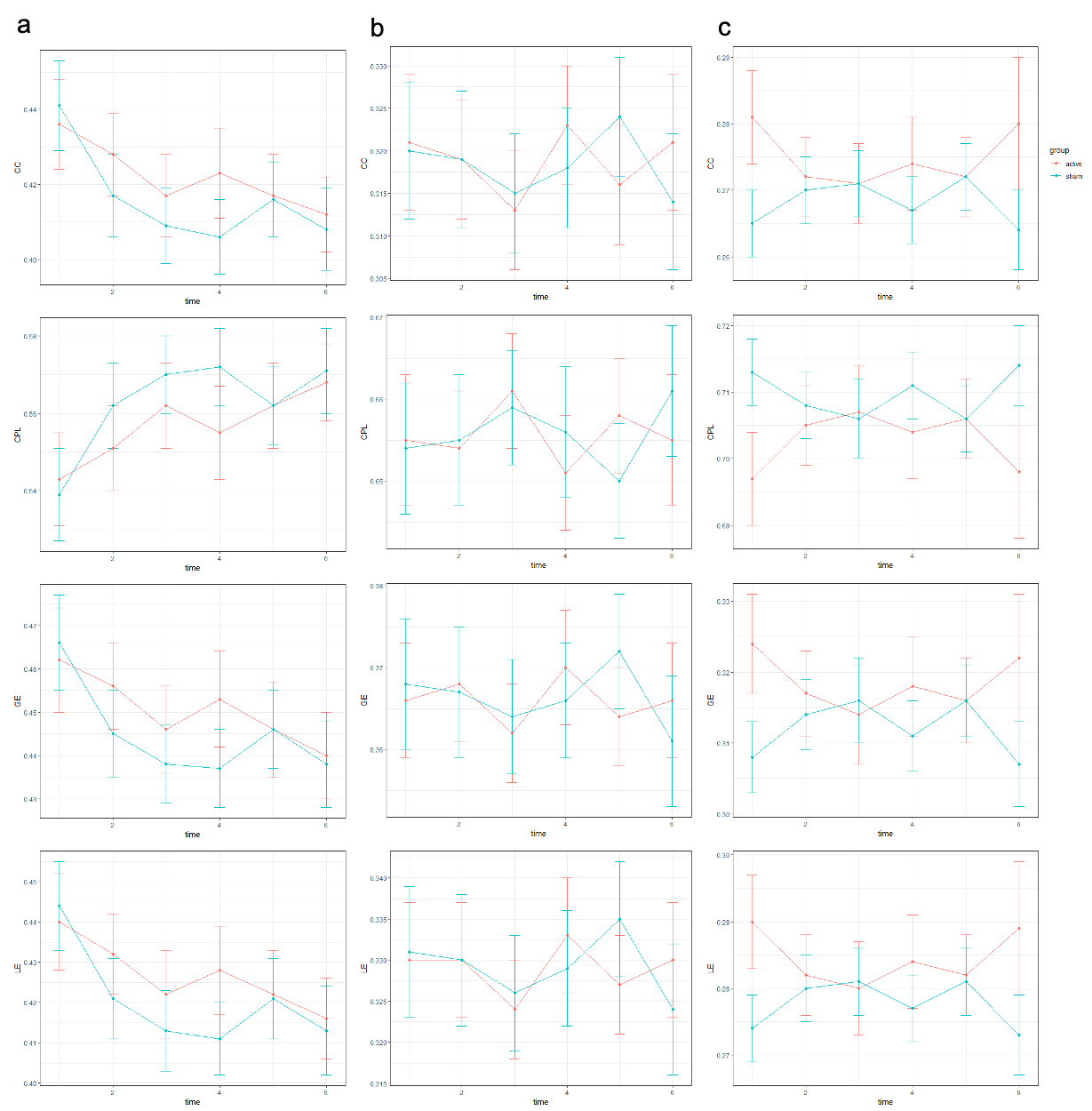
The statistical differences of network properties between two conditions in the alpha (a), beta (b), and gamma (c) band. The two-way repeated ANOVA was insignificant.

**Fig. S5. The power spectral density between pre and post-cTBS of each session.**


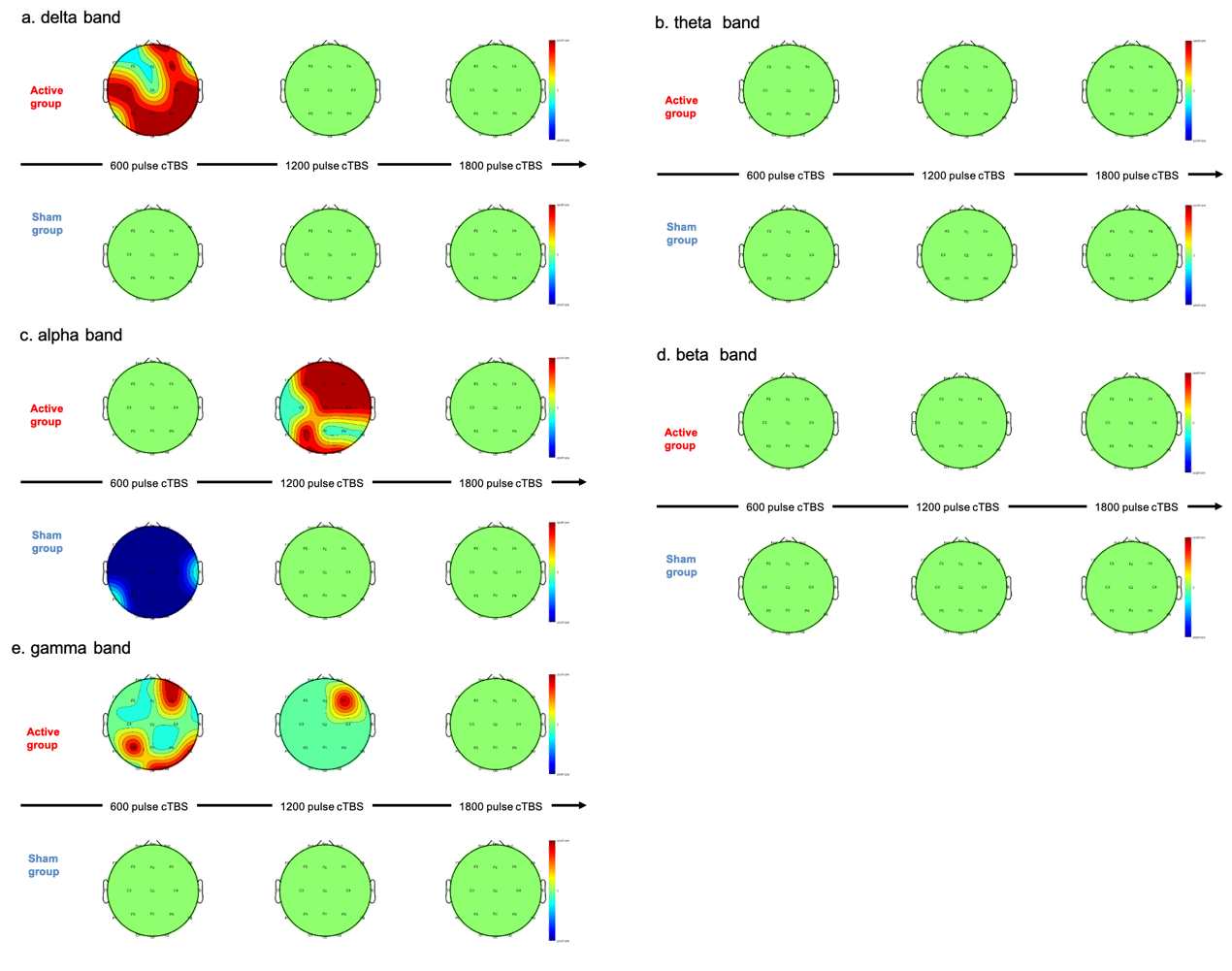


The topological distribution of power spectral density (PSD) of delta (a), theta (b), alpha (c), beta (d), and gamma (e) bands were compared between *pre* and *post*- cTBS of each session.

**Fig. S6. The power spectral density and phase locking value of delta and theta bands during the NREM of the first sleep cycle**

**
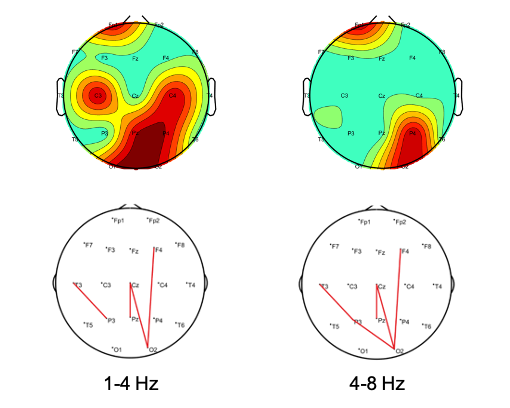
**

The topological distribution of spectral power of delta and theta band after active cTBS was higher in the occipital area than the sham condition in NREM of the first sleep cycle.
